# The Prevalence of Peyronie's Disease in the United States: A Population-Based Study

**DOI:** 10.1371/journal.pone.0150157

**Published:** 2016-02-23

**Authors:** Mark Stuntz, Anna Perlaky, Franka des Vignes, Tassos Kyriakides, Dan Glass

**Affiliations:** 1 Deerfield Institute, New York, New York, United States of America; 2 Formerly at Deerfield Institute, New York, New York, United States of America; 3 Yale Center for Analytical Services, Yale School of Public Health, New Haven, Connecticut, United States of America; University of Sydney, AUSTRALIA

## Abstract

Peyronie’s disease (PD) is a connective tissue disorder which can result in penile deformity. The prevalence of diagnosed PD in the United States (US) has been estimated to be 0.5% in adult males, but there is limited additional information comparing definitive and probable PD cases. We conducted a population-based survey to assess PD prevalence using a convenience-sample of adult men participating in the ResearchNow general population panel. Respondents were categorized according to PD status (definitive, probable, no PD) and segmented by US geographic region, education, and income levels. Of the 7,711 respondents, 57 (0.7%) had definitive PD while 850 (11.0%) had probable PD. Using univariate logistic regression modeling, older age (18–24 vs 24+) (OR = 0.721; 95% CI = 0.570,0.913), Midwest/Northeast/West geographic region (South vs Midwest/Northeast/West) (OR = 0.747; 95% CI = 0.646,0.864), and higher income level (<25K vs 25K+) (OR = 0.820; 95% CI = 0.673,0.997) were each significantly associated with reduced odds of having a definitive/probable PD diagnosis compared with no PD diagnosis. When all three variables were entered in a stepwise multivariable logistic regression, only age (OR = 0.642; 95% CI = 0.497, 0.828) and region (OR = 0.752; 95% CI = 0.647, 0.872) remained significant. This study is the first to report PD prevalence by geographic region and income, and it advocates that the prevalence of PD in the US may be higher than previously cited. Further, given the large discrepancy between definitive PD cases diagnosed by a physician and probable cases not diagnosed by a physician, much more needs to be done to raise awareness of this disease.

## Introduction

Peyronie’s disease (PD), described by and named after Francois Gigot de la Peyronie, is a localized connective tissue disorder that arises from plaque formation, caused by the deposition of collagen and fibrin in the tunica albuginea of the penis [1,2]. This fibrous plaque replaces the normally elastic fibers and can result in penile deformity. The disease is characterized by an initial or acute inflammatory phase which usually lasts about 12–18 months where the clinical hallmarks are unstable penile deformity and pain on erection. The stable or chronic phase begins when the acute phase subsides and it is characterized by stable penile deformity [3].

Patients with PD usually present within 6 months of symptom onset, and the earliest presenting symptom of PD is typically penile curvature [4]. Other common presenting symptoms are penile pain and penile plaque [5].

The etiology of PD has not been fully elucidated, but one hypothesis is that PD is a disorder of wound healing [6]. Further, trauma has been proposed as another inciting factor [7]. Interestingly, Dupuytren’s contracture (DD), a fibro proliferative condition of the palmar fascias in the hand, typically resulting in progressive contracture of one or more fingers, has been associated with PD [8]. The association between the two diseases was first recognized in 1828 and reported by Abernethy [9]. DD is thought to be the most common hereditary connective tissue disorder in Caucasians and according to the literature there is a 3–15% chance that a man with DD will have PD [10–13]. In one study of 415 male subjects with PD, 89 (22.1%) also had DD and a total of 28 men (6.7%) reported to have one or more first or second degree relatives with PD [14].

PD diagnosis is based on medical history, symptomatology, and identification of penile plaque by palpation. In some instances, imaging can also be used to identify the site and characteristics of the plaque [15]. Compared with older adults, young men are more likely to present at an early stage in the disease process, to have diabetes, and to have more than one plaque at the time of presentation [16–17].

Even though several prevalence studies have been carried out worldwide, estimates of the prevalence of PD in the general population remain limited and variable [18–19]. This is because studies have been carried out in different age groups and in different subpopulations of men such as older men undergoing prostate cancer screening, men with diabetes mellitus, or men with erectile dysfunction, rather than in men in the general population [20–25]. Moreover, varying definitions of PD were used to ascertain prevalence in published reports.

There are only three population-based epidemiological cross-sectional studies that have addressed the prevalence of PD; two of these studies were carried out in the United States (US) and one in Germany. The first cross-sectional study of PD was published in 1991 by Lindsay et al [20]. This study was carried out in Rochester, Minnesota, using the Mayo Clinic’s centralized medical records linkage system and the reported prevalence was 388.8 per 100,000 population (0.39%). Based on results from this study, it was estimated that there were 423,000 men with PD in the US at that time, and that 2,000 new cases occur annually. The mean patient age at diagnosis was 53 years with a range of 19 to 83 years of age [26].

More recently, a web-based survey of a large (n = 11,420) probability-based panel of research subjects representative of the full US population estimated the prevalence of PD to range from 0.5% (the percentage of surveyed subjects with PD diagnosis) to 13% (percentage with diagnosis, treatment, or penile symptoms of PD) [27].

The German study from Sommer et al (2002) provided the first PD prevalence estimates in Europe. This study sent a validated questionnaire survey to 8000 men ages 30–80 years in the greater Cologne region; a total of 142 (3.2%) of the respondents (n = 4,432) reported palpable plaque, which, from the previous validation, was the most sensitive question and the main symptom of the disease. The prevalence of the disease was 1.5, 3.0, 4.0, and 6.5% for men ages 30–39 years, 40–59 years, 60–69 years, and greater than 70 years, respectively [28].

Our study utilized a web-based survey conducted among the general US population of men ages 18 years or older. The study’s objectives were to: 1) estimate the prevalence of probable and definitive PD cases; 2) estimate the prevalence of PD symptoms among probable and definitive cases; 3) ascertain treatment and/or surgical interventions used for PD cases; and 4) assess whether associations exist between prevalence of PD and geographic region, age, or income level.

## Materials and Methods

### Survey design

A cross-sectional, population-based survey was conducted among a convenience-sample of men in the US ages 18 years and older. Participants from the ResearchNow general population panel were blinded to the survey topic before agreeing to participate in order to minimize potential selection bias. Respondents were recruited to an online survey and screened for current, self-reported symptoms of PD, the severity of and treatment or surgical intervention for said symptoms, and a self-reported past or present PD diagnosis by geographic region, age, and income level [29].

ResearchNow maintains a panel of individuals available for survey recruitment. The ResearchNow general population panel consists of more than three million consumers and is broadly representative of the US population in terms of age, gender, and region. Panelists have opted to participate in web-based survey research; in exchange for their participation, panelists receive points that are redeemable for tangible goods. Informed consent was provided through the opt-in process and the action of choosing to participate in a given survey. To maintain participant confidentiality, all identifying information was managed by the panel vendor and blinded to the survey administrator. As a member of CASRO (Council of Survey Research Organizations), the main professional body overseeing survey-based market research, ResearchNow adheres to the code of ethics mandated by this organization, which protects individual survey-based research subjects [30]. ResearchNow independently collects and maintains demographic information on their panel respondents, with the ability to append respondent-level data post-field.

The sample for this study was randomly selected from the ResearchNow general population panel of men ages 18 years and older, accounting for discrepancies in geography and time zone. To be representative of the general population, the sample was deployed in batches, controlling for age categories based on the stratification of males’ ages in the 2010 US census.

### Ethics, consent and permissions

This study was approved by the Deerfield Institute Research Review Committee. The major function of this committee is to ensure that survey research participant identities are protected, and that survey research proposed by the Deerfield Institute meets the highest scientific and ethical standards. For this study, the committee deemed that the survey research was conducted in accordance with the Code of Federal Regulations, Title 45, Department of Health and Human Services (DHHS), Part 46, Protection of Human Subjects [31]; that the survey research was conducted in accordance with ethics practices outlined by CASRO; and that the study was in full compliance of HIPAA (Health Insurance Portability and Accountability Act) guidelines, as it did not collect protected private health information that could be used to identify participants. Respondents were offered an industry-standard honorarium for their time to complete the survey. By opting into the survey, the responders provided consent to use their anonymized responses to the survey questions.

### Survey Content

The survey administered to participants assessed the current presence of PD symptoms, with those having symptoms answering questions whether they have discussed symptoms with a physician, how bothersome the symptoms were perceived to be, and whether they prevented successful intercourse. Additional questions addressed surgical intervention or the application of other treatment types to correct the shape of the penis, as well as the specific indication leading to these treatments. For those undergoing surgery or injections, respondents answered whether the treatments resulted in symptom relief. Lastly, the survey contained questions regarding a past or current PD diagnosis. The survey content was based on the survey used by DiBenedetti et al (2011) [28].

Demographic information not addressed in the survey including geographical region and income were provided and appended to the dataset by ResearchNow.

### Selection of Participants

The survey was active in the field from August 15 to October 22, 2014. To qualify for the survey, respondents had to specify their gender (male) and their age (at least 18 years). A total of 7,711 men ages 18 years and older completed the survey. Table 1 demonstrates that the sample was broadly representative of the US population and that the composition of those who responded did not differ meaningfully from the larger ResearchNow panel.

**Table 1:** Demographics: US Census (2010) and survey sample.

Demographic	Category	US Census	Panel	Survey Sample	Sample vs Census	Sample vs Panel
		%	%	%	p	p
Age (years)	18–24	13.1	10.0	9.8	0.02	0.88
	25–34	17.5	24.0	15.8	0.31	0.000006*
	35–44	17.5	21.0	16.9	0.72	0.02
	45–54	19.2	19.0	21.4	0.22	0.18
	55–64	15.6	16.0	14.1	0.35	0.23
	65–74	9.3	8.0	11.4	0.12	0.01
	75+	7.9	2.0	10.5	0.044	0.0000001*
Region	Midwest	21.7	22.0	24.4	0.15	0.20
	Northeast	17.9	19.0	20.4	0.16	0.43
	South	37.1	35.0	31.1	0.005*	0.064
	West	23.3	24.0	24.2	0.64	0.92
Income	<$25K	24.9	12.2	13.6	0.00001*	0.35
	$25K to <50K	25.2	18.9	21.2	0.034	0.20
	$50K to <$75K	18.1	21.1	23.5	0.003*	0.20
	$75K to <100K	11.5	16.7	18.5	0.0001*	0.29
	$100K to <$150K	11.9	17.8	14.7	0.07	0.06
	$150K+	8.2	13.3	8.6	0.75	0.0008*

* Statistically significant p-value based on chi-square test with Bonferroni correction.

### PD Case definitions

For analysis purposes, PD cases were determined using a combination of responses to survey questions and a physician diagnosis (self-reported):

*Definitive PD*: Responses to survey questions indicative of PD **AND** a PD diagnosis was provided by the participant’s physician.
*Probable PD*: Responses to survey questions indicative of PD **BUT NO** PD diagnosis provided by the participant’s physician.
*No PD*: Responses to survey questions not indicative of PD and no PD diagnosis provided by the participant’s physician.

When comparing with survey participants who were classified as ‘No PD’ cases, the two classifications of PD cases were combined into one level (Definitive/Probable).

For categorical variables, analyses and comparisons were performed using chi-square methods. Bonferroni corrections were used when multiple comparisons were performed, resulting in the *α* level being reduced to *α*/n. For all other analyses, a p-value <0.05 was considered statistically significant. Parametric (t-test and ANOVA) methods were used to compare continuous variables as needed and appropriate. The associations between various survey factors and PD status (Probable/Definitive PD vs No PD) were assessed.

Univariate and multivariable (stepwise) logistic regression models were used to generate Odds Ratios (with 95% CI) of PD status using various demographic characteristics as independent predictors. All statistical analyses were performed using SAS 9.3 (SAS Institute, Cary NC).

### Ethics Statement

The survey was conducted online between August 15 to October 22, 2014. Respondents were offered points redeemable for tangible goods for their time in completing the questionnaires. All survey data were analyzed in the aggregate and the individual identities of survey respondents were blinded to the study authors. The study did not collect protected private health information and was IRB exempt. All survey research conducted for this article was done in accordance with the ethics practices outlined by the Council of American Survey Research Organizations (CASRO).

## Results

7,711 men were recruited to complete the self-screening survey tool. Table 1 presents the demographic characteristics for the survey sample, the larger ResearchNow panel, and for the entire US population for men ages 18 years and older. The survey sample is well aligned with the Census distribution with respect to age and by region. While the survey sample is more heavily concentrated in higher income groups (a common bias in online survey research), this pattern is consistent between the sample survey and the broader ResearchNow panel, thus suggesting similarity between responders and non-responders across this dimension.

The mean age of all study participants was 48.8 years (SD = 17.6) while the mean age of those who received a definitive PD diagnosis was 57.9 years (SD = 18.1) and the mean age of those who had a probable PD diagnosis was 48.3 years (SD = 18.8) (Table 2).

**Table 2:** PD prevalence by demographic subgroups.

	No PD	Probable PD	Definitive PD	PD Prevalence (Net)	Total
Total n (%)	6804 (88.2)	850 (11.0)	57 (0.7)	907 (11.8)	7711
Age (years) n (%)					
18–24	640 (85.0)	111 (14.7)	2 (0.3)	113 (15.0)	753
25–34	1064 (87.1)	151 (12.4)	6 (0.5)	157 (12.9)	1221
35–44	1178 (90.5)	116 (8.9)	7 (0.5)	123 (9.5)	1301
45–54	1510 (91.3)	136 (8.2)	8 (0.5)	144 (8.7)	1654
55–64	948 (87.0)	131 (12.0)	11 (1.0)	142 (13.0)	1090
65–74	764 (86.9)	106 (12.1)	9 (1.0)	115 (13.1)	879
75+	700 (86.1)	99 (12.2)	14 (1.7)	113 (13.9)	813
Mean (#)	48.8	48.3	57.9	48.9	48.8
Region n (%)					
Midwest	1693 (90.4)	166 (8.9)	14 (0.7)	180 (9.6)	1873
Northeast	1384 (88.6)	171 (10.9)	7 (0.4)	178 (11.4)	1562
South	2055 (86.2)	309 (13.0)	20 (0.8)	329 (13.8)	2384
West	1649 (88.8)	191 (10.3)	16 (0.9)	207 (11.2)	1856
Income n (%)					
<$25K	843 (86.0)	132 (13.5)	5 (0.5)	137 (14.0)	980
$25K to <50K	1336 (87.4)	188 (12.3)	5 (0.3)	193 (12.6)	1529
$50K to <$75K	1493 (87.8)	193 (11.4)	14 (0.8)	207 (12.2)	1700
$75K to <100K	1188 (89.1)	133 (10.0)	13 (1.0)	146 (10.9)	1334
$100K to <$150K	933 (87.9)	116 (10.9)	12 (1.1)	128 (12.1)	1061
$150K+	561 (90.3)	53 (8.5)	7 (1.1)	60 (9.7)	621

Table 2 provides the prevalence of definitive, probable, and total PD across all survey respondents and among the demographic sub-categories of region, age, and income. Of the 7,711 respondents, 57 (0.7%) were categorized as definitive PD cases while an additional 850 (11.0%) were categorized as probable PD cases. With a total of 907 definitive and probable cases among 7,711 respondents, we estimated a total PD prevalence of 11.8%.

In univariate logistic regression analyses, age, region, and income were independently significant predictors of Probable/Definitive PD. Respondents who were attributed a Probable/Definitive PD diagnosis were: (a) less likely to have been in the 24+ age group (OR = 0.721; 95%CI = 0.57,0.913); (b) less likely to be in non-South regions of the country (OR = 0.747; 95% CI = 0.646,0.864; (c) less likely to be in the $25K income level (OR = 0.820; 95% CI = 0.673, 0.997).

When all three variables (age, region, income) were entered in a stepwise multivariable logistic regression, only age and region remained significant. Respondents who were attributed a Probable/Definitive PD diagnosis were: (a) less likely to have been in the 24+ age group and (b) less likely to be in non-South regions of the country (Table 3).

**Table 3:** Predicting probable/definitive PD versus no PD diagnosis.

	OR	95% CI	p
***Univariate models***			
Age (18–24 vs 24+)	0.721	0.570, 0.913	0.0066
US Region (South vs Midwest/Northeast/West)	0.747	0.646, 0.864	<0.0001
Income Level (<25K vs 25K+)	0.820	0.673, 0.997	0.0469
Multivariable model (stepwise)*			
Age (18–24 vs 24+)	0.642	0.497, 0.828	0.0006
US Region: South vs Midwest/Northeast/West	0.752	0.647, 0.872	0.0002

* All three variables in model.

Table 4 shows that there were significant differences (p<0.0001) between definitive PD cases and probable PD cases with respect to the occurrence of four symptoms: (a) unusual firmness or hardened tissue under non-erect penis; (b) lump/bump, firmness/hardness affecting shape of erect penis; (c) development of a new or significant curve or bend of erect penis; and (d) indentation on one or both sides of erect penis, with definitive PD cases having a significantly higher proportion of cases with the above-mentioned symptoms.

**Table 4:** Symptoms and treatment.

	Probable PD	Definitive PD	PD Net	p
Total	850	57	907	
	n (%)	n (%)	n (%)	
QS4X1: Lump or bump under skin of non-erect penis				0.86
No	635 (74.7)	42 (73.7)	677 (74.6)	
Yes	215 (25.3)	15 (26.3)	230 (25.4)	
QS4X2: Unusual firmness or hardened tissue under non-erect penis				<0.0001
No	734 (86.4)	37 (64.9)	771 (85.0)	
Yes	116 (13.6)	20 (35.1)	136 (15.0)	
QS4X3: Lump/bump, firmness/hardness affecting shape of erect penis				<0.0001
No	758 (89.2)	29 (50.9)	787 (86.8)	
Yes	92 (10.8)	28 (49.1)	120 (13.2)	
QS4X4: Development of a new or significant curve or bend of erect penis				<0.0001
No	597 (70.2)	24 (42.1)	621 (68.5)	
Yes	253 (29.8)	33 (57.9)	286 (31.5)	
QS5X1: Indentation on one or both sides of erect penis				<0.0001
No	739 (86.9)	36 (63.2)	775 (85.5)	
Yes	111 (13.1)	21 (36.8)	132 (14.5)	
QS5X2: Noticeable narrowing of erect penis				0.22
No	697 (82.0)	43 (75.4)	740 (81.6)	
Yes	153 (18.0)	14 (24.6)	167 (18.4)	
QS5X3: Erect penis folds during intercourse				0.4
No	496 (58.4)	30 (52.6)	526 (58.0)	
Yes	354 (41.6)	27 (47.4)	381 (42.0)	
Current PD symptoms				<0.0001
No	6 (0.7)	8 (14.0)	14 (1.5)	
Yes	844 (99.3)	49 (86.0)	893 (98.5)	
Ever had PD treatment				<0.0001
No	821 (96.6)	46 (80.7)	867 (95.6)	
Yes	29 (3.4)	11 (19.3)	40 (4.4)	
QS6X1: Surgery for PD treatment				0.001
No	822 (96.7)	49 (86.0)	871 (96.0)	
Yes	28 (3.3)	8 (14.0)	36 (4.0)	
QS6X2: Injections for PD treatment				<0.0001
No	810 (95.3)	43 (75.4)	853 (94.0)	
Yes	40 (4.7)	14 (24.6)	54 (6.0)	

With respect to PD treatment, 19.3% of definitive PD cases and 3.4% of probable PD cases had treatment that was specific for PD (p<0.0001) (Table 3).

## Discussion

Estimating the prevalence of PD presents a challenge as patients may hesitate to discuss their symptoms with a physician. Moreover, prevalence estimates may differ by age, geographic location, comorbidities, and socioeconomic status. Studies estimating the prevalence of PD have yielded variable results due in part to studies being carried out in different subpopulations as well as different settings, such as population/community or hospital/clinic [19, 23–25]. For general populations, Schwarzer et al (2001) and Sommer et al (2002) found a prevalence of 3.2% in males ages 30–80 years in Cologne, Germany, while Lindsay et al (1991) found a prevalence of 0.39% in males ages 19 years and older in a US study conducted in Rochester, Minnesota from 1950–1984 [20,26,27]. More recently, DiBenedetti et al (2011) found a prevalence of 13% among males ages 18 years and older and this estimate included males who had been diagnosed, treated, or currently had penile symptoms [28]. The variability in prevalence estimates from these studies is due in part to investigators using different definitions. These studies add valuable insight, but the lack of a consistent use of definitions make inter- and intra-country comparisons between studies impossible. To this end, we undertook this cross-sectional study using identical definitions used in the survey published by DiBenedetti et al (2011). We opted to use DiBenedetti’s wording rather than basing our survey on the Peyronie’s Disease Questionnaire (PDQ), used in the Investigation for Maximal Peyronie’s Reduction Efficacy and Safety Studies (IMPRESS) [32]. The PDQ was developed to quantify the psychosexual impact of PD, and we felt that for the purposes of a general population-based epidemiological study, it was more appropriate to base our survey on language previously used to measure the epidemiological burden of PD, which allowed us to compare and contrast our results with one of the few published studies investigating the prevalence of PD. In addition, we expanded on these previously published estimates by including key demographic information to our findings. To the best of our knowledge, this study is the first to segment the prevalence of PD in the US by geographical region, age, and income.

Our findings indicate that the prevalence of definitive and probable cases of PD in the US is 0.7% and 11% respectively. Our estimate of 0.7% for definitive (diagnosed) cases is similar to the 0.5% prevalence of diagnosed cases found by DiBenedetti et al (2011). Moreover, we estimated a combined prevalence of 11.8% for both probable and definitive cases while DiBenedetti et al (2011) estimated a prevalence of 13% among the same population.

Among the strengths of our cross-sectional study is the use of a population-based sample of adult males recruited via an online survey. As the survey was conducted online, participants may have been more likely to share confidential information about PD symptoms, diagnosis, and treatment. In addition, the participants were stratified by geographic region, age, and income. In hindsight, inclusion of data on marital/relationship status and race may have been useful to attempt to capture.

A limitation of the current study is the use of self-report data from participants. Despite a process allowing for confidentiality, respondents may have been uncomfortable in answering the questions truthfully. The survey questions also omitted penile pain as a symptom, which may be the first or only symptom that a patient mentions to a physician. Additionally, the wording of the questionnaire may have inadvertently captured patients with conditions similar to, but distinct from, PD, including distal type hypospadias and fibroproliferative conditions such as Dupuytren’s contracture, although these conditions are quite rare. Participants with the uncommon condition of congenital penile curvature may also have been captured by the questionnaire wording, although the disclaimer “compared to when you were younger…” was used in select questions in order to minimize this potential confounding. The definitions used for definitive and probable PD are not universally accepted; however, they have been used in previously published research on PD [28]. Finally, the online survey methodology utilized in this study restricts the amount and type of data that can be collected, although it is the most appropriate study design for a general population epidemiological survey.

The difference in prevalence between definitive and probable cases, 0.7% and 11.0%, respectively, suggests that PD may be under- and/or misdiagnosed by physicians who are not familiar with the condition or that existing screening tools for PD are inadequate. The difference in the prevalence between definitive and probable cases may also be due to patients being hesitant to visit a physician about their condition. Interestingly, our findings suggest that men 25 years and older are less likely to have probable or definitive PD compared with men 18–24 and that respondents in the South compared with respondents from the Midwest/Northeast/West were more likely to have a probable or definitive PD diagnosis. These findings are thought provoking, but further research will be required to strengthen these conclusions and elucidate the basis for these findings.

The difference between the mean age of probable cases and mean age of definitive cases suggests that there may be a time lag between the onset and stabilization of symptoms and diagnosis by a physician. Notably, DiBenedetti et al (2011) estimated the mean age of respondents who had received a diagnosis or treatment was 59.6 years (SD = 11.9) and 49.6 years (SD = 17.0) for respondents who had symptoms, but no diagnosis/treatment. There were significant differences (p<0.0001) between definitive PD cases and probable PD cases with respect to the occurrence of the four main symptoms. This is important as it points to those symptoms that would be most useful for physicians to diagnose PD in patients and would be critical for physicians to refer to when screening for PD.

Population-based, cross sectional studies provide epidemiological data on the prevalence of disease that are useful for increasing awareness, implementing screening strategies that may result in expediting treatment, and assessing potential effectiveness of interventions.

This study, the first to report PD prevalence by geographic region and income, suggests that the prevalence of PD in the US may be higher than previously cited [20]. Further, given the prevalence of probable cases not diagnosed by a physician, much more needs to be done to raise awareness of this disease.

## Supporting Information

S1 File Peyronie’s Disease questionnaire. (DOCX)
