# Supplementary material for: The Prevalence of Peyronie's Disease in the United States: A Population-Based Study
Source: PLoS One. 2016 Feb 23;11(2):e0150157. doi: 10.1371/journal.pone.0150157 (PMC4764365; doi:10.1371/journal.pone.0150157)
Supplement: S1 File — (DOCX) [file pone.0150157.s001.docx]

**Deerfield Institute**

**PD Epi Survey**

**Revision Date: 09-03-14**

**Category Definitions / Map**

**Diagnosed:** QS6x2 = YES

**Treated:** (QS6x1= YES OR QS6x2= YES) AND (QS10x4=YES OR QS10x5=YES)

**Symptomatic:** QS4x1=YES OR QS4x2=YES OR QS4x4=YES OR QS5x1=YES OR QS5x2=YES OR QS5x3=YES

| **SCREENER** |
| --- |

**BASE: ALL RESPONDENTS**

**S1**. Please indicate your:

**Age:**

___ years

[RANGE: 9-99][TERMINATE IF <18]

**BASE: ALL RESPONDENTS**

**S4**. Do you currently have any of the following symptoms?

|  | **Yes** | **No** |
| --- | --- | --- |
| 1. Lump or bump under the skin of non-erect penis (not including genital warts, pimples, or blisters) | **◦** | **◦** |
| 1. Unusual firmness or hardened tissue under the skin of non-erect penis (not including genital warts, pimples, or blisters) | **◦** | **◦** |
| 1. Lump/bump, firmness, or hardened tissue affecting the shape of erect penis | **◦** | **◦** |
| 1. Development of a new significant bend or curve of erect penis | **◦** | **◦** |

**BASE: ALL RESPONDENTS**

**S5**. Compared to when you were younger, do you have any of the following changes in your erect penis? *Please select all that apply.*

1. An indentation has developed on one or both sides of penis
2. Noticeable narrowing of penis
3. Penis folds during sexual intercourse
4. Penis is shorter than it used to be
5. The head of penis is less hard than it used to be
6. None of the above [EXCLUSIVE]

**BASE: HAS SYMPTOMS (S4/1-4=YES OR S5/1-3)**

**S9**. You indicated that you have the following symptoms:

[DISPLAY RESPONSES SELECTED AT S4/1-4 AND S5/1-3 AS BULLETED POINTS]

1. Have you ever specifically discussed these symptoms with a physician?
2. Yes
3. No
4. How bothersome are these symptoms to you currently?
5. Not at all bothersome
6. Somewhat bothersome
7. Very bothersome
8. Do these symptoms prevent you from having successful intercourse?
9. Yes
10. No

**BASE: ALL RESPONDENTS**

**S6**. Have you ever had any of the following treatments to correct the shape of your penis?

|  | **Yes** | **No** |
| --- | --- | --- |
| 1. Surgery | **◦** | **◦** |
| 1. Injections | **◦** | **◦** |
| 1. Topical treatments | **◦** | **◦** |
| 1. Other (specify: ___) | **◦** | **◦** |

**BASE: HAD SURGERY OR INJECTIONS (S6/1=YES OR S6/2=YES)**

**S10**. Which of the following best describes your reason(s) for having surgery or injections to correct the shape of your penis? *Please select all that apply.*

1. Erectile dysfunction
2. Circumcision
3. Penile implant or enlargement
4. Correct curve or bend when erect
5. Treat bump, lump, or plaque under the skin when not erect
6. Other (specify: ____________)

**BASE: HAD SURGERY OR INJECTIONS FOR PD SYMPTOM (S10/4-5)**

**S11**. You indicated that you had sugery/injections to correct a curve or bend or to treat a bump, lump, or plaque under the skin of your penis. What was the result of this treatment?

1. The symptoms went away and have not returned
2. I still have the symptoms or the symptoms have since returned

**BASE: ALL RESPONDENTS**

**S7**. Have you ever been diagnosed by a physician with any of the following conditions?

|  | **Yes** | **No** |
| --- | --- | --- |
| 1. Prostate cancer | **◦** | **◦** |
| 1. Peyronie’s disease | **◦** | **◦** |
| 1. Erectile dysfunction | **◦** | **◦** |
| 1. Testicular cancer | **◦** | **◦** |
| 1. Chordee or congenital curvature of the penis | **◦** | **◦** |

**BASE: BEEN TOLD THEY HAVE PEYRONIES (S7/2=YES)**

**S8**. Do you currently have a diagnosis of Peyronie’s Disease?

1. Yes
2. No
